# Oral Vaccination of Baculovirus-Expressed VP28 Displays Enhanced Protection against White Spot Syndrome Virus in *Penaeus monodon*

**DOI:** 10.1371/journal.pone.0026428

**Published:** 2011-11-01

**Authors:** Syed Musthaq S, Jimmy Kwang

**Affiliations:** 1 Animal Health Biotechnology, Temasek Lifesciences Laboratory, National University of Singapore, Singapore, Singapore; 2 Department of Microbiology, Faculty of Medicine, National University of Singapore, Singapore, Singapore; INRA, France

## Abstract

White Spot Syndrome Virus (WSSV) is an infectious pathogen of shrimp and other crustaceans, and neither effective vaccines nor adequate treatments are currently available. WSSV is an enveloped dsDNA virus, and one of its major envelope proteins, VP28, plays a pivotal role in WSSV infection. In an attempt to develop a vaccine against WSSV, we inserted the VP28 gene into a baculovirus vector tailored to express VP28 on the baculovirus surface under the WSSV ie1 promoter (Bac-VP28). The Bac-VP28 incorporated abundant quantity (65.3 µg/ml) of VP28. Shrimp were treated by oral and immersion vaccination with either Bac-VP28 or wild-type baculovirus (Bac-wt). The treatment was followed by challenge with WSSV after 3 and 15 days. Bac-VP28 vaccinated shrimp showed significantly higher survival rates (oral: 81.7% and 76.7%; immersion: 75% and 68.4%) than Bac-wt or non-treated shrimp (100% mortality). To verify the protective effects of Bac-VP28, we examined *in vivo* expression of VP28 by immunohistochemistry and quantified the WSSV copy number by qPCR. In addition to that, we quantified the expression levels shrimp genes LGBP and STAT by real-time RT-PCR from the samples obtained from Bac-VP28 vaccinated shrimp at different duration of vaccine regime. Our findings indicate that oral vaccination of shrimp with Bac-VP28 is an attractive preventative measure against WSSV infection that can be used in the field.

## Introduction

With a high economic output—US$ 9.0 billion annually—shrimp culture plays a major role in global aquaculture [1]. The successful production of shrimp is hampered by several viral diseases, particularly White Spot Syndrome Virus (WSSV). It has been one of the most threatening infectious pathogens to the shrimp culture industry over the past two decades. WSSV-related cumulative mortality typically reaches 100% within 2 to 5 days of the onset of clinical signs. The virus has a wide host range that includes freshwater prawns, lobsters, freshwater crabs and several species of marine crabs [2], [3], [4], [5]. WSSV belongs to the genus *Whispovirus* (www.ncbi.nih.gov/ICTVdb/Ictv/index.htm) under the viral family *Nimaviridae*. It is a rod-shaped, enveloped dsDNA virus approximately 275 nm in length and 120 nm in width with tail-like appendages at one end. Sequencing of the full genome of WSSV revealed that it has 184 ORFs, but the role of many of its viral proteins is still unknown. As of this study, 39 structural proteins have been identified in WSSV, including 22 envelope proteins [6]. The WSSV genome contains at five known major structural proteins: VP28, VP19, VP26, VP24 and VP15. Studies on WSS viral proteins have demonstrated that VP28 and VP19 are associated with the virion envelope. VP26 acts as a tegument protein linking the two nucleocapsid-associated proteins VP24 and VP15 to the envelope [7].

Novel viral control strategies, including vaccination, are necessary due to the continued high growth of shrimp cultivation and the broad host range of WSSV. Invertebrates lack a true adaptive immune response and rely solely on an innate immune response, so until recently, vaccination of shrimp against WSSV was not considered a viable strategy. However, Venegas et al. [8] demonstrated the existence of quasi-immune response in *Penaeus japonicus* that had survived previous exposure to WSSV. In another study, shrimp became resistant to WSSV about 3 weeks after infection and hemolymph from resistant shrimp contained virus neutralizing factors [9]. These results stimulated research into the possibility of vaccinating shrimp against WSSV. Within a few years, numerous published studies explored different WSSV vaccination strategies to protect shrimp, including inactivated WSSV vaccines [10], [11], subunit recombinant vaccines [12], [13], [14], [15], oral recombinant vaccines [16], [17], DNA vaccines [18], [19], [20] and dsRNA [21], [22].

In most WSSV vaccines, VP28 has been a major target, as the envelope structural proteins of WSSV play a pivotal role in initial viral infection. The envelope proteins are often necessary for viral budding, entry and assembly. VP28 is a major envelope protein of WSSV and is involved in the systemic infection of shrimp [23], [24]. During WSSV infection, VP28 interacts with host cellular proteins such as PmRab7 [25], heat shock cognate protein 70 [26] and the signal transducer and activator of transcription (STAT) [27] to bring about the viral infection. Previous reports have demonstrated that VP28-based recombinant vaccines provide protection and increase the survival rate of challenged shrimp during WSSV experiments when compared with unvaccinated shrimp [10], [15], [16], [17]. However, the protection offered by these VP28 derived recombinant vaccines is not high, and the protective response is drastically low at 10 days post-challenge. It may be possible to improve vaccine efficacy by expressing the recombinant VP28 protein in a baculovirus eukaryotic expression system.

In the present study, we inserted VP28 gene into baculoviral vector under the control of the WSSV ie1 promoter and expressed VP28 gene (Bac-VP28) on baculovirus surface. Our quantitative western blotting technique showed, abundant quantity of VP28 was expressed on Bac-VP28. Next, we demonstrate for the first time an efficacious immunisation of WSSV immersion-challenged shrimp by administering Bac-VP28 via oral and immersion routes. The protective response generated in Bac-VP28 immunized shrimp against WSSV was measured by quantification of WSSV viral copy numbers by quantitative real-time PCR (qPCR) and *in vivo* expression of VP28 by immune histochemistry (IHC). To authenticate vaccine efficacy we quantified shrimp Lipopolysaccharide and β-1,3-glucan binding protein (LGBP) and STAT genes expression profiles by real-time RT-PCR from the samples obtained from vaccinated shrimp at different duration of vaccine regime. Hence, Bac-VP28 is an attractive preventative measure for shrimp culture against WSSV infection that can be used in the field applicable approach.

## Materials and Methods

### Collection and maintenance of experimental animals

Shrimp, *Penaeus monodon* (10–12 g body weight), were imported from Malaysia and maintained in 1000-l fiberglass tanks with air-lift biological filters at an ambient temperature of 27–30°C with salinity between 20 and 25 ppt. Natural seawater was used in all the experiments. It was pumped from the adjacent sea to Singapore and allowed to sediment to remove the sand and other suspended particles. The seawater was then chlorinated by treating with sodium hypochlorite at the concentration of 25 ppm and then dechlorinated by vigorous aeration, before being passed through a sand filter and used for the experiments. The animals were fed with artificial pellet feed (BTA feed, Malaysia). Temperature and pH were recorded, salinity was measured with a salinometer (Aquafauna, Japan) and dissolved oxygen was estimated by the Winkler method. The animals were kept in tanks for 5 days and acclimatized prior to the experiments. From the experimental animals, 5 from a group of 30 were randomly selected and screened for the presence of WSSV by polymerase chain reaction (PCR) using the primers designed by Takahashi et al. [28] and only healthy shrimp were used for the experiments.

### Preparation of WSSV viral inoculum

The laboratory WSSV stock was used in this study and it was propagated by injecting into healthy shrimp. The hemolymph was drawn directly from the hearts of moribund shrimp using sterile syringes followed by centrifugation (3000× g for 20 min at 4°C). The supernatant fluid was then re-centrifuged (8000× g for 30 min at 4°C) and the final supernatant fluid was filtered through a 0.4 µm filter. The filtrate was then stored at −80°C for experimental studies.

### Generation of recombinant baculovirus

For the construction of VP28 gene into pFASTBacHT A (life technologies, USA) baculovirus transfer vector contains polyhedrin promoter including His tag, ATG and multiple cloning sites was replaced with WSSV ie1 promoter with *RsrII* and *HindIII* restriction enzymes. The ie1 promoter was amplified from WSSV DNA using the primers WSSVie1F-5′-CCTACGTATCAATTTTATGTGGCTAATGGAGA-3′and WSSVie1R-5′-CGCGTCGACCTTGAGTGGAGAGAGAGCTAGTTATAA-3′ then inserted into pFASTBacHT A using *AccI* and *RsrII* restriction site. The full length ORF of VP28 gene was amplified from WSSV genome using the primers Bac-VP28F 5′-CGCCGGTCCGATGGATCTTTCTTTCACTCTTTC-3′ and Bac-VP28R 5′-CCGAAGCTTTTACTCGGTCTCAGTGCCAG-3′ with *RsrII* and *HindIII* restriction enzymes and cloned into modified pFASTBacHT A vector.

For the generation of recombinant baculoviruses the constructs were integrated into the baculovirus genome within DH10Bac™ (life technologies, USA) through site-specific transposition according to the protocol of Bac-to-Bac system (life technologies). The recombinant baculoviruses with VP28 gene was named as Bac-VP28 and without VP28 gene was known as Bac-wt. Further recombinant baculovirus were propagated in SF-900II SFM (life technologies) at 27°C and infecting Sf9-cells as described by Syed Musthaq et al. [29].

### Immunofluorescence assay to detect the expression of VP28 in insect cells

To detect the Immunofluorescence signals, Sf9 cells were grown in 24 well plates and infected with Bac-VP28 and Bac-wt baculovirus at a MOI of 0.5. After 48 hrs post infection, the cells were fixed with paraformaldehyde for 20 min and blocked with 1% gelatin for 30 min at room temperature. The fixed cells were then incubated with anti-mouse PrVP28 (prokaryotic expressed recombinant VP28) polyclonal antibody prepared by Syed Musthaq et al. [29] at a dilution of 1∶100 for 1 hr at 37°C. FITC-conjugated rabbit anti-mouse (DakoCytomation, Denmark) at a dilution of 1∶100 was subsequently incubated with the cells for 1 hr. The fluorescence signal was detected with an inverted fluorescence microscope (Olympus, UK) and the images were captured by a digital imaging system (Nikon, USA).

### Western blot analysis of VP28

For Western blot analysis, the Bac-VP28 infected cells or supernatant were mixed with Laemmli sample buffer and SDS-PAGE was carried out as described by Laemmli [30]. VP28 from purified WSSV virions served as a reference while Bac-wt was included as a negative control. The gel was transferred to nitrocellulose membrane and Western blot was performed by the method of Talbot et al. [31]. The anti-mouse PrVP28 polyclonal antibodies at a dilution of 1∶2000 were used as primary antibody and rabbit anti-mouse IgG (DakoCytomation, Denmark) at a dilution of 1∶1000 were used as secondary antibody to detect the baculovirus expression of VP28.

### Quantitative western blotting

Odyssey Infrared Imager (LI-COR, Biotechnology) was used to calculate the amount of VP28 present on recombinant baculovirus. The PrVP28 expressed in bacterial system and purified with His-tag fusion partner was used as a standard. The anti-mouse VP28 polyclonal antibodies at the dilutions of 1∶2000 and a dilution of 1∶10,000 of donkey anti-mouse IRdye800CW IgG (LI-COR, Biotechnology) was used as primary and secondary antibody respectively. The membrane was developed and band intensities were analysed by Odyssey Application software version 1.2. The amount of VP28 displayed on Bac-VP28 was calculated by comparing with PrVP28 as standard control.

### Vaccination experiment and WSSV challenge

#### Oral vaccination

For the oral vaccination experiment, commercial shrimp pellet feed weighing 2 g were coated with 3 ml of 1×10^8^ pfu/ml of baculovirus containing Bac-VP28, Bac-wt and positive, negative control pellets were coated with phosphate buffer saline (PBS). The feed was mixed and incubated on ice for 30 min followed by room temperature (RT) incubation for 30 min to allow absorption. The pellets were coated with fish oil to prevent dispersion of the baculovirus or PBS in water. A batch of shrimp was divided into four groups and 20 shrimps per group were selected. The shrimp in group first and second were administrated orally with Bac-VP28 and Bac-wt coated feed continuously for 7 days, whereas group third and fourth were administrated orally with PBS coated feed. Third day after final vaccination, the shrimp in group one to three were immersed in a sea water containing a dilution of 1∶150 WSSV stock solution for 2 hrs, then the shrimp was changed in to fresh seawater without WSSV, whereas group four was challenged with PBS containing seawater. Another batch of shrimp (4 groups, 20 shrimp each group), the vaccine experiment was repeated and shrimp were challenged with WSSV on 15 days post vaccination (dpv), whereas group 4 was challenged with PBS. The oral vaccination experiments were repeated three times.

#### Immersion vaccination

In the vaccination experiment, a batch of shrimp was divided into four groups and 20 shrimps per group were selected. The shrimp in group first and second were immersed in 2 liters of seawater containing 3 ml 1×10^8^ pfu/ml of Bac-VP28 and Bac-wt respectively for 2 hr, whereas the third and fourth groups were immersed in seawater containing 1 ml of PBS. The immersion vaccination was repeated another 2 times at 5 day intervals. Third day after final vaccination, the shrimp in group one to three were immersed in a sea water containing a dilution of 1∶150 WSSV stock solution for 2 hrs, then the shrimp was changed in to fresh seawater without WSSV, whereas group four was challenged with PBS. In another batch of shrimp (4 groups, 20 shrimp each group), the vaccine experiment was repeated and shrimp were challenged with WSSV on 15 dpv, whereas group 4 was challenged with PBS. The immersion vaccination experiments were repeated three times.

### *In vivo* expression of VP28

Immuno histochemistry analysis was performed to evaluate the *in vivo* expression of VP28 at the translational level. The shrimp tissues (eye stalk and hepatopancrease) were collected from Bac-VP28 and Bac-wt vaccinated shrimp at 7 dpv and was fixed in 10% buffer formalin. After 48 hr, the samples were processed and embedded in paraffin wax using Embedder (Leica, Germany), then sectioned into 4 µm thickness using microtome (Leica Autocut microtome model 2255, Leica Microsystems, Wetzlar, Germany). The sections were de-paraffinized using Histo-choice (Amersco, Solon, Ohio, USA) and rehydrated in sequentially graduated ethanol baths to distilled water. The sections were treated with trypsin (0.1% w/v in PBS) for 10 min and washed twice with PBS-Tween 20 (0.01% v/v with PBS). Slides were blocked in 0.1% non fat milk in PBS for 30 min followed by incubation with anti-mouse PrVP28 polyclonal antibody at a dilution of 1∶100 for 1 h at room temperature. Slides were then washed 3 times in PBS-T and incubated with HRP-conjugated rabbit anti-mouse (DakoCytomation, Denmark) at a dilution of 1∶50 for 30 min. After washing, the sections were mounted using glycerol. The slides were observed under a microscope (Olympus, UK) and the images were captured by digital imaging system (Nikon, USA).

### Quantification of WSSV by real-time PCR

Quantitative real-time PCR was performed to determine the WSSV viral loads in shrimp tissues samples collected at 2, 5, 7, 10 and 15 days of post infection (dpi). The viral copy number was estimated by using Rotor Gene Q (Qiagen Inc., USA) using DyNAmo™SYBR® Green qPCR Kit (Finnzymes, Espoo, Finland). Briefly, the primers QVP26F-5′-ATCTCTACCGTCACACAGCC-3′ and QVP26R-5′-GAAGATTTTAATGTCCTTGCTCG-3′ was used to amplify a 211-bp fragment from the VP26 gene of WSSV. Viral DNA was isolated from 50 mg of gill tissue by using the QIAamp DNA tissue kit (Qiagen Inc., USA) according to the manufacturer's instructions. DNA was resuspended in 50 µl of DNase-RNase-proteinase-free water. The PCR parameters consisted of 40 cycles of denaturation at 94°C for 10 sec, annealing at 58°C for 5 sec, and extension at 72°C for 10 sec. A standard curve was obtained using serial dilutions of plasmid pVP26 (full length ORF of VP26 gene of WSSV was cloned into pQE-30 vector) were used to quantify the WSSV viral genomic copy number. The geometric mean of viral genomic copies per reaction was calculated for each group after setting results for negative samples to one copy per sample. Each assay was carried out in triplicate.

### Quantification of LGBP and STAT genes expression by real-time RT-PCR

Real-time RT-PCR was carried out to quantify the mRNA expressions of shrimp LGBP and STAT genes from healthy or WSSV infected and Bac-VP28 vaccinated shrimp's hepatopancreas tissue collected at 2, 5, 7, 10 and 15 dpi. Briefly, the total RNA was extracted by Trizol method following the manufacturer's protocol (life technologies, USA) and RNA quality and quantity was determined by Nanodrop spectrophotometer ND-1000. DNA contamination was removed by using RNase free DNase I (Fermentas Inc, USA) and synthesis of cDNA was performed with oligo dT primer (Roche, USA) and cDNA conversion mix (Promega, USA) as per manufacturer's instructions. The amplifications were performed by using Rotor Gene Q (Qiagen Inc., USA) real time PCR machine using DyNAmo™SYBR® Green qPCR Kit (Finnzymes, Espoo, Finland) with cDNAs of respective genes. The highly gene specific quantitative real-time RT-PCR primers targeting LGBP gene [32] and STAT gene [33] were used to analysis the expression levels of LGBP and STAT genes in experimental shrimp tissues. Briefly, the following primers were used in this study, for LGBP gene (289-170F-5′GGTAACCAGTACGGAGGAACGA′3), (289-233R -5′TACTCGACGTGGGTCTTCTCGA′3) and for STAT gene (390F- 5′AGCCCCTGTCTGAGCGAAA′3), (461R- 5′GGTGTTCTCTTGTGACCTTCATCA′3). For all real-time assays, internal control gene, shrimp EF-1α (elongation factor) specific primers were used and described by Roux et al. [32]. The thermal profile for SYBR Green RT-PCR was 50°C for 2 min and 95°C for 10 min followed by 40 cycles of 95°C for 15 s and 60°C for 1 min. The dissociation curve of each PCR product was a single peak and each sample was run in triplicate along with an internal control gene. The SYBR Green assay was repeated three times independently. The ΔΔCt method was used to monitor the transcription level of shrimp LGBP and STAT target genes. The Ct values of amplified target genes (Ct_LGBP_ & Ct_STAT_) and internal control gene (Ct_EF-1α_) in each sample were computed by relative quantification method described in Rotor Gene Q. ΔCt values were then calculated by subtracting Ct_EF-1α_ from Ct_STAT_ or Ct_LGBP_. To normalize the data, ΔCt for each tested samples (Ct_STAT_ or Ct_LGBP_) was subtracted from the average ΔCt of the calibrator (PBS- injected to shrimp), to produce a value called ΔΔCt. The change in transcription level of each sample relative to the transcription level of the calibrator was then expressed as 2^−ΔΔCt^. The data obtained from this real time PCR were subjected to a T-test where p-values below 0.05 were considered to be significant.

### Statistical analysis

The values are expressed as arithmetic mean ± SD. The level of significance was expressed as P<0.01. The protection against WSSV after vaccination was calculated as the relative percent survival (RPS) (1-mortality of vaccinated group/mortality of control group)×100 [34].

## Results

### Generation of recombinant baculovirus and its confirmation

**Figure 1:**
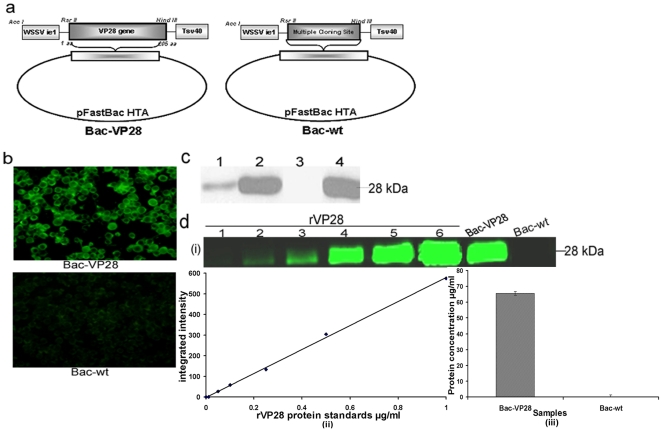
Schematic structure of recombinant baculovirus construct, analysis the expression of VP28 on baculovirus system using anti-mouse VP28 polyclonal antibodies and Quantification of VP28 expressed from Bac-VP28. (a) The VP28 gene was cloned downstream of WSSV ie1 promoter and upstream of Tsv40 of pFasBacHT vector and named as Bac-VP28 and above construct with out VP28 gene named as Bac-wt. (b) Immunofluorescence assay of expression of VP28 in Sf9 cells. The cells were infected with Bac-VP28 and Bac-wt at MOI of 0.5 and at 48 hrs post infection the cells fixed and analysed. (c) Western blot analysis of baculovirus expressed VP28 compared with WSSV virions. Lane 1-supernatant of baculovirus expressed VP28; Lane 2-complete Bac-VP28 virions; Lane 3-complete Bac-wt virions; Lane 4-purified wild type WSSV virions from WSSV infected shrimp tissue. (d) (i) -quantitative western blot analysis of VP28 expressed in baculovirus system and compared with different concentration of PrVP28 expressed in bacterial system as a standard. Lane 1 to Lane 6- purified PrVP28 protein from 0.01 µg, 0.05 µg, 0.1 µg, 0.25 µg, 0.5 µg and 1 µg; Lane Bac-VP28-1 µg total protein; Lane Bac-wt-1 µg of total protein. The nitrocellulose membrane was scanned and analysed by Odyssey Infrared Imager. (ii)-rVP28 protein standard curve; (iii)-the amount of VP28 protein presents in Bac-VP28 culture.

The WSSV genome encoding envelope protein VP28 was selected for the generation of recombinant baculovirus with an immediate early promoter 1 (ie1) derived from the WSSV genome and named as Bac-VP28 (Fig. 1a). The above construct without VP28 gene is termed as Bac-wt (Fig. 1a). The recombinant baculoviruses are generated in Sf9-II cells and followed the method described by Bac-to-Bac expression system (life technologies, USA). The expression of VP28 was analyzed by Immunoflourescence assay and results indicated that baculovirus counter part VP28 expressed in insect cells and maintains its structural and antigenic conformity (Fig. 1b). Next, we compared the molecular mass of VP28 present in the baculovirus infected cell lysate or complete virion and purified WSSV virions by the western blot analysis, which detected a band both with the similar size of 28 kDa (Fig. 1c) with anti-mouse PrVP28 polyclonal antibody. However, neither fluorescent signal nor specific product was detected in Bac-wt derived from insect cells or lysate.

### Quantitative western blotting

For the quantification of VP28 displayed on Bac-VP28, we carried out quantitative western blotting technique using Odyssey Infrared Imager (LI-COR, Biotechnology). The VP28 protein concentration presents in Bac-VP28 was compared with PrVP28 expressed in *E.coli* system as standard control and results were analysed by Odyssey Application software version 1.2. From the calculation, the amount of VP28 presents in Bac-VP28 was measured on the average of 65.3 µg/ml (10^8^ pfu) obtained from three independent batches of baculovirus culture (Fig. 1d).

### Vaccination experiment and WSSV challenge

#### Oral vaccination

**Figure 2:**
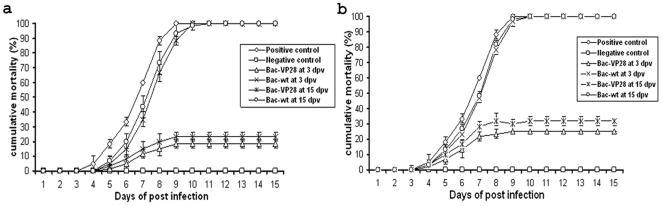
Time-mortality relationship of (a) oral and (b) immersion vaccinations and WSSV challenge experiment. Shrimp were vaccinated with Bac-VP28, Bac-wt or PBS (positive control) and challenged with WSSV or PBS buffer (negative control) on 3 and 15 days post vaccination (dpv).

In this experiment, the efficacy of surface displayed recombinant baculovirus (Bac-VP28) was tested through oral administration method. Two batches of shrimp contain four groups each (20 shrimp per group) were selected as indicated in Table 1. The shrimp in batch I, (group 1, and 2) was administrated orally with Bac-VP28 and Bac-wt coated feed for 7 days continuously and group 3 received PBS. After vaccination, the shrimp in group 1 to 3 of batch I was subsequently challenged with a WSSV by immersion route at 3 dpv. Whereas, the same groups of shrimp in batch II was administrated with Bac-VP28 or Bac-wt or PBS coated feed for 7 days and challenged with WSSV at 15 dpv. Experimental shrimp were observed twice a day and the resulting time-mortality relationship is shown in Fig. 2a. A significantly lower cumulative mortality of 18.3% and 23.3% (relative survival of 81.7% and 76.7% ) was observed in Bac-VP28 vaccinated shrimp at 3 dpv and 15 dpv respectively, when compared to positive control or Bac-wt group showed a cumulative mortality of 100% at 9 to 10 dpi. Whereas the negative control groups showed no mortality throughout the experimental period. These results are consistent with an independent experiment, resulting Bac-VP28 vaccinated group showed significantly higher survival rate compared Bac-wt or positive control group.

**Table 1:** Set up for vaccination experiments.

Experiment Name	Groups	Vaccine/Coating	No. of shrimp/group
Oral vaccination	Bac-VP28	Baculovirus expressed VP28	20×3
	Bac-wt	wild type baculovirus	20×3
	Positive control	PBS	20×3
	Negative control	PBS	20×3
Immersion vaccination	Bac-VP28	Baculovirus expressed VP28	20×3
	Bac-wt	wild type baculovirus	20×3
	Positive control	PBS	20×3
	Negative control	PBS	20×3

#### Immersion vaccination

In this experiment, the potential of surface displayed recombinant baculovirus was tested immersion method. Two batches of shrimp contain four groups each (20 shrimp per group) were selected as indicated in Table 1. The shrimps in batch I (group 1 and 2) were given through immersion with Bac-VP28 and Bac-wt and group 3 received PBS. After vaccination, the shrimp in group 1 to 3 of batch I was subsequently challenged with a WSSV by immersion route at 3 dpv. Whereas, the same groups of shrimp in batch II was administrated with Bac-VP28 or Bac-wt or PBS and challenged with WSSV at 15 dpv. Experimental shrimp were observed twice a day and the resulting time-mortality relationship is shown in Fig. 2b. A significantly lower cumulative mortality of 25% and 31.6% (relative survival of 75% and 68.4%) was observed in Bac-VP28 vaccinated shrimp at 3 dpv and 15 dpv respectively, when compared to positive control or Bac-wt group showed a cumulative mortality of 100% at 9 to 10 dpi. Whereas the negative control groups showed no mortality throughout the experimental period. These results are consistent with an independent experiment, and Bac-VP28 vaccinated group showed significantly higher survival rate compared Bac-wt or positive control group.

### *In vivo* expression of VP28 in shrimp

**Figure 3:**
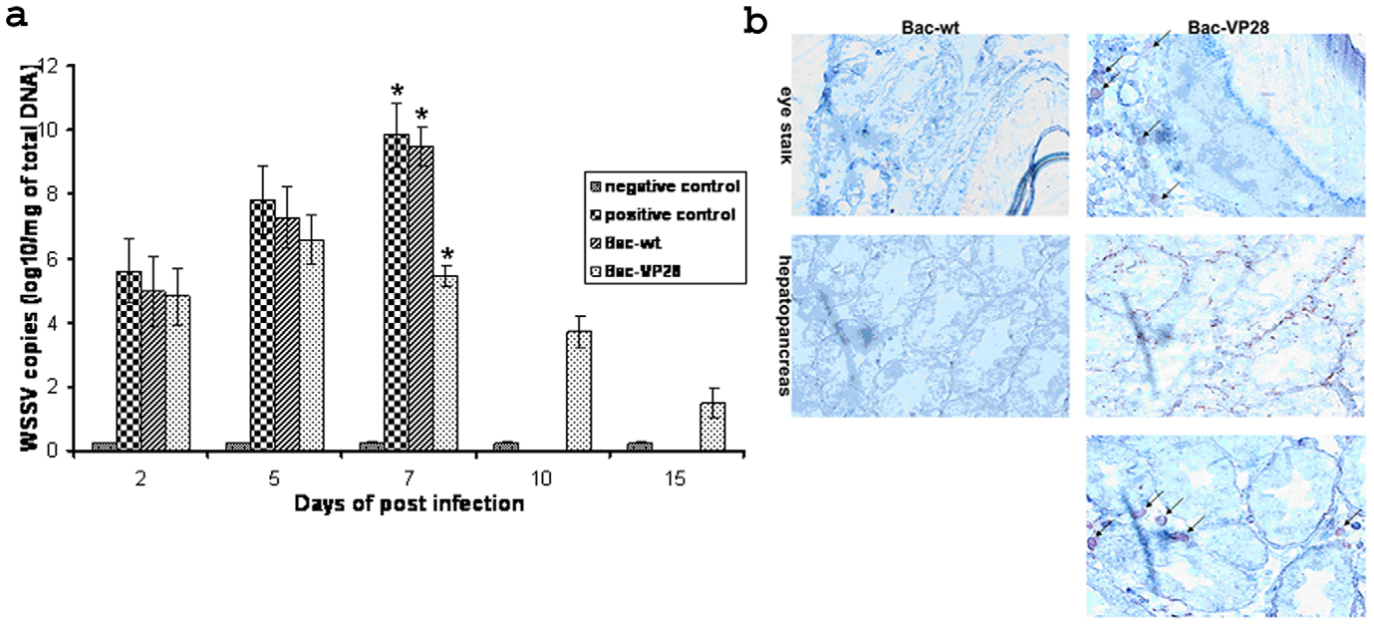
Analysis of the protection generated by recombinant baculovirus by (a) quantification of WSSV viral copies by real-time PCR (b) immunohistochemistry analysis of *in vivo* expression of VP28 from Bac-VP28 and Bac-wt vaccinated shrimp tissues. In Figure a, each column represents the mean of triplicate assay with standard deviation. Asterisks denote significant differences (P<0.01) between samples. All shrimp in the 10 dpi and 15 dpi samples of Bac-wt, Positive control group were died and data is Not Detectable. In Figure b, the arrow marks indicates the expression of VP28 in shrimp tissues.

To analysis *in vivo* delivery capacity of WSSV ie1 based Bac-VP28 or Bac-wt baculoviruses and expression of VP28 measured by immunohistochemistry from the organs of vaccinated shrimp at 7 dpv. The results concealed that the Bac-VP28 was transduced effectively and the expression of VP28 protein was observed in the shrimp tissues eyestalk and hepatopancreas at 7 dpv (Fig. 3b).

### Quantification of WSSV by real-time PCR

The WSSV infected organs from the groups of vaccinated shrimp were collected for the quantification of WSSV viral copy numbers by SYBR green PCR at different days post infection. The results revealed that, viral copy numbers were increased in the groups of shrimps till 5 dpi, later the copy numbers in Bac-VP28 shrimp was significantly reduced to 5.44×10^10^ WSSV copies mg−1 on 7 dpi, then dropped to 1.36×10^10^ WSSV copies mg^−1^ on 15 dpi (Fig. 3a). Whereas the viral copy numbers in Bac-wt or positive control groups reached its peak level of 9.89×10^10^ WSSV copies mg^−1^ and more on 9 dpi and later the shrimp were died. Statistically significant difference has been observed at 7 dpi. There is no viral copy numbers or change has been observed in negative control group in experimental period.

### Quantification of LGBP and STAT genes expression by real-time RT-PCR

**Figure 4:**
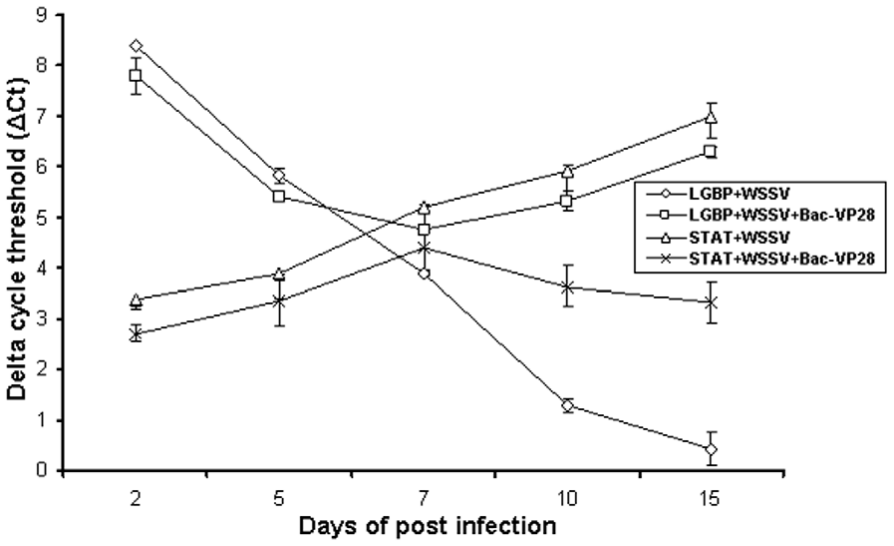
Temporal expression analysis LGBP and STAT genes in healthy or WSSV infected shrimp and compared with Bac-VP28 vaccinated shrimp at 2, 5, 7, 10 and 15 dpi. The relative expression level was expressed by ΔCt (Ct value of LGBP/STAT gene-Ct value of EF-1α gene) and determined for each samples. The average ΔCt for WSSV infected or vaccinated samples from each days post infections was then used to plot the graph.

Real-time RT-PCR was used to quantify, the changes in expression levels shrimp LGBP and STAT genes obtained from the tissues of normal shrimp or WSSV infected shrimp and compared with Bac-VP28 vaccinated shrimp tissues at different dpi. Data were expressed as ΔCt and changes in the transcription levels in LGBP and STAT genes of Bac-VP28 vaccinated relative to WSSV infected shrimp was shown in Fig. 4. The expression level of STAT gene in WSSV control group downregulated in various dpi samples as infection progresses, whereas vaccinated group showed decreasing trends of STAT gene till 7 dpi and changes to increasing level as infection by WSSV low due to recombinant baculoviruses. The values at 10 dpi and 15 dpi were statistically significant. The expression levels of LGBP gene in WSSV group upregulated as infection progress, whereas, vaccinated group showed increasing trend at early infections and slows down as WSSV infection cleared by Bac-VP28. Statistically significant difference was observed, where LGBP gene expression was decreases in vaccinated group at 7 dpi and 10 dpi (Fig. 4).

## Discussion

In the current study, we demonstrate for the first time an efficacious immunisation of WSSV immersion-challenged shrimp by administering Bac-VP28 via oral and immersion routes. In a previous study, we described how vaccination with a recombinant baculovirus surface displaying VP28 (Bac-VP28) elicited a significantly higher survival rate as compared with other forms of vaccination [29]. We chose the immersion method because the challenge pressure can be well controlled, and it is the natural infection mechanism in shrimp culture. The VP28 gene was inserted into a baculoviral vector under the control of the WSSV ie1 promoter (Fig. 1a). Previously, the ie1 promoter has been shown to have efficient activity in *Spodoptera frugiperda* (Sf9-II) insect cells, and the ie1 promoter is also known to act as shuttle promoter between insect and mammalian cells [35]. The ie1 promoter is active during the early stage of baculovirus infection of insect cells. The expression of VP28 was analysed by immunofluorescence assay, and the results indicated that VP28 is expressed in the insect cells and maintains both its structural and antigenic conformity (Fig. 1b). Interestingly, western blot analysis revealed that both baculovirus-expressed VP28 and the VP28 purified from WSSV virion particles migrated with similar sizes in SDS-PAGE (Fig. 1c). The result indicates that the posttranslational modification of VP28 in insect cells is similar to what occurs in shrimp cells. In addition, we demonstrated that VP28 is present on the baculovirus surface during its budding process and is incorporated into the baculovirus envelope [29]. Next, we quantified the amount of VP28 displayed on Bac-VP28 and was calculated by comparing with PrVP28 standard control. The results showed that VP28 in Bac-VP28 was on the average 65.3 µg/ml (10^8^ pfu) from three independent batches of baculovirus culture (Fig. 1d) and concluded that, abundant quantity of VP28 is expressed on the surface of the Bac-VP28.

In the present vaccination experiment, Bac-VP28 derived vaccination conferred significantly higher survival rates of 81.7% and 76.7% (cumulative mortality of 18.3% and 23.3%) on shrimp challenged at 3 and 15 dpv, respectively, by oral vaccination (Fig. 2a), and a survival rate of 75% and 68.4% (cumulative mortality of 25% and 31.6%) on shrimp challenged at 3 and 15 dpv, respectively, by immersion vaccination (Fig. 2b). The positive control and Bac-wt group showed a cumulative mortality of 100% at 9 to 10 dpv, whereas the negative control groups showed no mortality throughout the experimental period. These results are consistent with results we obtained in a separate experiment of the vaccination regimen in which the Bac-VP28 vaccinated group had a significantly higher survival rate than either the Bac-wt or positive control groups. Inferring from the VP28 quantitation, each shrimp consumed approximately 10.6 µg of VP28 from Bac-VP28 coated feed during the course of vaccination experiment. The quantity of VP28 delivered in this study correlates with previous subunit vaccination experiments with purified rVP28 against WSSV [15], [18], but the protection rate generated by recombinant baculovirus vaccination is significantly higher. The other studies on oral vaccination with rVP28 expressed in bacteria followed by inactivation, as vaccine candidate lacks rVP28 quantitation data [16], [17].

The Bac-VP28 vaccine demonstrated higher efficacy than bacterially expressed VP28 protein vaccination [16], [17], administration of DNA vaccines encoding the VP28 gene [19], [20] or bacterially expressed VP28 dsRNA administration [21], [22]. Shrimp fed with bacterially over-expressed VP28-coated feed demonstrated 64% and 77% relative survival when challenged on 3 and 7 dpv, but the protection was reduced significantly after 14 dpv [16]. In another study, crayfish fed orally with rVP28 protein-coated feed had a cumulative mortality of 43.2% at 3 dpv [17]. These studies also indicated that the maximum protection time conferred by a recombinant protein vaccine synthesised from bacteria was only in the first 2 weeks post-vaccination. For crayfish immunised orally with a *Salmonella typhimurium* encapsulated DNA vaccine plasmid encoding the VP28 gene, there was a survival rate of 66.7% at 15 dpv [20]. Rajeshkuamr et al. [19] demonstrated a 65% survival rate at 15 dpv for shrimp vaccinated with a chitosan nanoparticle encapsulated DNA vaccine plasmid encoding the VP28 gene. Sarathi et al. [22] observed a 68% survival rate for shrimp fed with bacterially expressed VP28 dsRNA-coated feed and challenged with WSSV 5 dpv. However, the practical application of such DNA-based vaccines or dsRNA to aquaculture species is limited, as they have been found to be effective only when delivered via gene gun, scarification [36] or, in the case of DNA vaccines, short-pulse ultrasound [37]. When the results of the present study are compared, the immersion vaccine method is more expensive, and the generated pathogen protection rate is lower than what is observed following than oral vaccination. Therefore, oral vaccination is the more convenient and inexpensive method, and it may be the only practical way to deliver potential vaccines to the cultured aquatic animals. Hence, the ability of baculovirus displaying VP28 to elicit an increased protection rate in vaccinated shrimp makes baculovirus a better vaccine candidate than other current forms of WSSV vaccination. Also, we considered to choose oral delivered Bac-VP28 shrimp tissues are optimum choice to monitor vaccine efficacy by other molecular assays.

To further verify the protection conferred by Bac-VP28, the WSSV viral copy numbers were quantified in oral vaccinated shrimp on different post-challenge days by quantitative real-time PCR. The results indicated that WSSV VP26 gene copy numbers increased in the Bac-VP28 groups until 6 dpi. The copy numbers observed in the Bac-VP28 shrimp were significantly reduced to 3.7×10^10^ WSSV copies mg^−1^ by 10 dpi and then dropped to 1.42×10^10^ WSSV copies mg^−1^ by 15 dpi (Fig. 3a). In contrast, the viral copy numbers in the Bac-wt and positive control groups reached a peak level of 9.89×10^10^ WSSV copies mg^−1^ and above of total DNA by 9 dpi before the shrimp succumbed to the virus. The virus was not observed in the negative control group during the experimental period. The viral copy numbers obtained in this study are similar to the observations of Durand and Lightner [38], who noted similar WSSV copy numbers (2.0×10^4^ to 10.2×10^10^ WSSV copies µg^−1^ of total DNA) after differing time intervals post-WSSV infection in shrimp. In the present study, the reduced viral copy numbers are possibly due to the activation of the innate antiviral immunity system in the vaccinated shrimp. Also, our immunohistochemistry results demonstrated that Bac-VP28 was transduced effectively and expressed VP28 in the eye stalk and hepatopancreas at 7 dpv (Fig. 3b). These results provide additional evidence for the Bac-VP28 vaccine's efficacy.

To support the vaccine efficacy generated by Bac-VP28, we purposed to study the host gene response in the experimental shrimp. There are increasing evidences postulated that defence mechanism of invertebrates elevated against invading pathogens. Pattern Recognition Proteins (PRP) are those defense mechanism molecules in invertebrates and plays major role to recognize and responds to infections. LGBP is one of the effective PRP, and responsible for the innate immune response in crustaceans and insects by activating prophenoloxidase (proPO) cascade [39], [40]. Roux et al. [32] compared expression level of LGBP gene in healthy and WSSV challenge shrimp in different hours of post infection. STAT is an another gene we chosen to study, because it play a vital role in the innate immunity of both vertebrates and invertebrates [41], [42], [43]. Janus Kinase (JAK)-STAT pathway involved in the antiviral defense mechanism in invertebrates [44], [45]. Also, Chen et al. [33] studied the STAT gene expressions in WSSV infected and PBS inoculated shrimp. So, we targeted to analysis LGBP and STAT genes expressions at transcriptional level in vaccinated shrimp challenged with WSSV using quantitative real-time RT-PCR. In the present, we observed LGBP gene was upregulated in WSSV infected shrimp proportionally to progress of the infection. But, in the Bac-VP28 vaccinated shrimp, the expression level of LGBP gene upregulated at early infections till at 7 dpi, later it downregulates at considerable level. At 10 dpi, a significant different was observed and WSSV infected shrimp showing higher expressions of LGBP gene than vaccinated group (Fig. 4). The LGBP gene expression level was statistically significant at (P<0.01) at 10 and 15 dpi. The STAT gene expression analysis in the present study demonstrated a similar level of expression patterns with earlier study [33] of WSSV infected shrimp. In the vaccinated shrimp, the expression trends is downregulated till 7 dpi, later it upregulated slowly and reaches significant level at 15 dpi (Fig. 4). The STAT gene expression level was statistically significant (P<0.01) at 10 and 15 dpi. As observed in this study, the changes in expression level in LGBP and STAT genes postulated that, the recombinant baculoviruses triggers innate immune response or efficiently decay the WSSV viral particles upon challenge as seen in quantification WSSV particles reduced at 15 dpi.

After examining the results of the present study, the WSSV protection generated in shrimp may be explained in three ways. First, the ability of ie1 promoter to transduce the VP28 efficiently in the shrimp tissue and to expresses VP28 protein for an extended duration may be involved. Similarly, Liu et al. [27], described that shrimp STAT enhances the expression of ie1 promoter. The promoter enhancement could indirectly activate the expression of the VP28 in the baculovirus construct. Second, the display of VP28 on the baculovirus surface theoretically makes it readily accessible for the earlier interactions with the shrimp cell and helps the cell to avoid further attachment of WSSV virions. A similar idea was proposed by Yang et al. [46] for hemagglutinin (HA), the viral envelope protein of influenza virus. The third explanation is that the persistent infection of shrimp by the recombinant baculovirus may reduce the severity of WSSV infection by an unknown mechanism. Such a result is part of the “viral accommodation” concept [47].

In summary, a novel approach was identified in which a recombinant baculovirus was used as a potential vaccine candidate for WSSV in shrimp. The ie1 promoter of WSSV was used to efficiently express VP28 consistently in insect cells, and the recombinant baculovirus is able to transduce and express VP28 successfully in shrimp cells without having any adverse effect on the shrimp. As observed in the present study, the oral administration of Bac-VP28 to shrimp generated a higher survival rate compared with immersion or other forms of vaccination. In addition, the good biosafety profile of the baculovirus as a vaccine vector in both humans and shrimp make it an attractive choice. Oral vaccination with recombinant baculovirus is an attractive choice for vaccination of shrimp against WSSV. The results of the present study open up new avenues in the development of novel field-applicable vaccines.
